# Supplementary material for: Compound Microalgae-Type Biofunctional Hydrogel for Wound Repair during Full-Thickness Skin Injuries
Source: Polymers (Basel). 2024 Mar 3;16(5):692. doi: 10.3390/polym16050692 (PMC10935078; doi:10.3390/polym16050692)
Supplement: Supplementary file 1 [file polymers-16-00692-s001.zip › polymers-2874209-supplementary.pdf]

Supporting information

Materials and methods

Synthesis of QCS

The amino group in chitosan reacts with GTMAC to generate quaternized chitosan (QCS). 0.5 g of chitosan was dissolved in 0.5% acetic acid solution and filtered out the insoluble substances. Deionized water was then added to dilute the filtrate three times and stirred for 30 min. GTMAC (6 μmol) was added dropwise, each titration time controlled within 30 min. The clear solution was obtained after reacting at 55 °C for 18 h. Then the insoluble were removed through centrifuging at 6500 rpm for 10 min at room temperature. Half of the above solution was distilled using water, and added to the pre-cooled acetone for precipitation. The precipitation process was repeated three times to produce a quaternary ammonium hydrocarbon glycan crude product. The crude product was then dissolved in water, and dialyzed in a dialysis membrane with MWCO of 8000-14000 against DDW for one week. The pure product was obtained by the freeze-dried technique. The residual amino and primary hydroxyl groups in quaternary ammonium hydro carbonate react with carboxyl groups in dihydrocaffic acid to generate HQCS. Specifically, solution containing 1.0% (*w*/*v*) QCS was prepared. Then, solution containing 1.0% (*w*/*v*) HCA and solution containing 1.0% (*w*/*v*) EDC (dissolved in DDW and ethanol (1:1, *v*/*v*)) were added dropwise into the above solution. Finally, the pH of the reaction mixture was adjusted to 5 with vigorously stirring at room temperature for 12 h. After that, the resulting solution was dialyzed (MWCO of membrane: 8000-14000) for one week. The final product was obtained by freeze-drying. FTIR, NMR, and UV-vis spectroscopy were used to characterize the structure.

Gelation behavior, self-healing behavior and injectability of the hydrogels

The storage modulus G' and the loss modulus G'' can be calculated by the following formula:

$$G' = \frac{\sigma}{\gamma}\cos\delta$$

$$G'' = \frac{\sigma}{\gamma}\sin\delta$$

Where δ is the phase angle, which depends on the response of the material. σ is the stress, γ is the strain. If the δ is zero, there is no hysteresis and the sample is an ideal elastic solid.

In vitro antibacterial properties of hydrogels

Briefly, target bacteria were cultured in Luria Broth (LB) medium at 37 °C with shaking at 200 rpm. Then, the old medium was removed by centrifugation; sediment was resuspended and diluted with fresh sterile medium, the number of bacterial suspensions was tuned to ~10^8 CFU mL^{-1}. Subsequently, 200 μL of different samples were added to a 96-well plate. Then 10 μL of the diluted bacterial solution was added to each well for co-cultivation for 6 h in a bacterial incubator at 37 °C. Sterile PBS was used as a control. Finally, 100 μL of the mixture was spread evenly on LB agar medium by 10-fold dilution method, which was standard at 37 °C for overnight culture. The number of CFU on the plate was counted with ImageJ software. The killing rate of the bacteria was calculated based on the formula:

$$\text{Kill ratio\%} = [(N_{control} - N_{sample})/ N_{control}] \times 100\%. \quad (1)$$

In addition, the bacterial suspensions treated in each group were centrifuged, fixed with glutaraldehyde, dehydrated with ethanol using different water contents, and sprayed with gold to observe the bacterial morphology by SEM (S-4800, Hitachi, Ltd., Japan). The SEM acceleration voltage used is 3 KV.

Moreover, we further studied the bacteriostatic ability of the hydrogel through the size of the inhibition zone. Specifically, the bacterial suspension was obtained according to the above

steps, infiltrated with a sterile cotton swab, and spread evenly on the LB agar plate to form a dense layer of flora. Samples with a diameter of 6 mm and a height of 1 mm were buckled from the cross-linked hydrogel with a 6 mm hole punch, and they were quickly placed in the center of the agar plate coated with bacterial liquid. The plate without hydrogel was used as the control group. The size of the zone of inhibition was measured after overnight incubation.

Antioxidant activity of hydrogels on chemical models

ABTS radical ($ABTS^{\cdot+}$) scavenging experiment: $ABTS^{\cdot+}$ scavenging activity is generally considered to be the total antioxidant capacity. ABTS was oxidized to green $ABTS^{\cdot+}$ under the action of appropriate oxidants, and the production of $ABTS^{\cdot+}$ was inhibited in the presence of antioxidants. Specifically, 2.45 mM solution of ABTS radicals dissolved in potassium persulfate solution was prepared, which was allowed to stand in the dark at room temperature for 16 h and diluted before use. The experiment was performed on a 96-well plate. The total volume of the solution to be tested was 200 μL, wherein the volume ratio of the sample solution and the ABTS solution was 1:19. Subsequently, it was left to stand in the dark for 15 minutes at room temperature. The absorbance of the resulting solution was measured at 734 nm by a microplate reader (Bio Tek CYTATION5 imaging reader, Agilent, America). The total antioxidant capacity is calculated as follows:

$$\text{Total antioxidant capacity (\%)} = [1-(A_s/A_c)] \times 100\% \quad (2)$$

where A_s represents the absorbance of the solution obtained without sample treatment, and A_c represents the absorbance of the solution obtained by treatment with ultrapure water.

DPPH radical scavenging experiment: First, 0.2 mM DPPH radical solution was prepared. The experiment was carried out in a 96-well plate, and the total volume of the solution to be tested was 200 μL, wherein the volume ratio of the sample solution and the DPPH solution was 1:4. Necessarily, the effect of the color of the sample itself needs to be deducted for each sample group. After being thoroughly mixed by shaking for 60 s and kept in the dark for 30 min, the absorbance of the resulting solution was measured at 517 nm by a microplate reader. The DPPH inhibition percent was recorded as the DPPH free radical scavenging performance of each sample, which was calculated as the following equation:

$$\text{DPPH inhibition (\%)} = [1-(A_s-A_b)/A_c] \times 100\% \quad (3)$$

where A_s represents the absorbance of the solution obtained without sample treatment, A_b represents the absorbance of the color of the sample itself, and A_c represents the absorbance of the solution obtained by ultrapure water treatment.

Hydroxyl radical scavenging experiment: The quantitatively added Fe^{2+} and H_2O_2 in the reaction system generate hydroxyl radicals through the Fenton reaction. Free radicals and oxidants oxidize a part of Fe^{2+} to Fe^{3+}, reducing the orange-red complex (Fe(phen)). Antioxidants are added to the system to react with free radicals and oxidants in the system so that the reduction of Fe^{2+} is inhibited. Specifically, the total volume of the solution to be tested is 200 μL. First, 1 volume of citric acid buffer (0.1M, pH 4.4), H_2O_2 (0.02%), ferrous sulfate solution (6 mM), and 3 volumes of ultrapure water were mixed and ·OH was generated by the Fenton reaction between them. Subsequently, 1 volume of sample solution was added to the solution to eliminate ·OH and the remaining ·OH was detected with phenanthroline (6 mM) as an indicator. In addition, $FeSO_4$, hydrogen peroxide and indicator were not added in the blank group, the test sample in the damaged group was the sample solvent, and the test sample in the undamaged group was the sample solvent without adding hydrogen peroxide, and ultrapure water was added to each group to ensure that the total system volume reached 200 μL. After 30 minutes in the dark at 37 °C, the absorbance of all sample solutions was measured at 510nm by an ultraviolet spectrophotometer, and the elimination percentage of ·OH was calculated as follows:

$$\cdot\text{OH Elimination (\%)} = [(A_s-A_c-A_0) / (A_1-A_0)] \times 100\% \quad (4)$$

where A_s represents the absorbance of the solution obtained without the sample treatment, A_c represents the absorbance of the color of the sample itself, A_1 represents the undamaged absorbance, and A_0 represents the damaged absorbance.

In addition, the full wavelength of the free radical scavenging reaction was scanned by a UV spectrophotometer (TU-1950, Beijing Spectroscopy General Instrument Co., Ltd).

Cytotoxicity test and Live/Dead staining

Mouse fibroblasts NIH-3T3 were seeded in a 96-cell well plate at ~1×10^5 cells/mL density and cultured in an incubator at 5% carbon dioxide and 37 °C for 24 h until the cell density reached about 90%. Subsequently, the old medium was removed and carefully washed twice with PBS. The hydrogel performs HQCS and SP were dissolved in DMEM, respectively, prepared into different concentrations of sample solutions, added to the cell well plate in a volume of 100 μL, and incubated in a cell incubator for 24 h. The sample solution was carefully removed, and an equal volume of MTT solution (0.02%) dissolved in DMEM was added. After incubating in the cell incubator for 4 h in the dark, carefully remove the MTT solution, add 100 μL of DMSO solution to each well, and shake at low speed with a microplate reader for 10 min to fully dissolve the crystals. Furthermore, measure the optical density (OD) at 490 nm. All samples were repeated 6 times (n = 6). Cell viability was calculated as follows:

$$\text{Cell viability (\%)} = (A_s/A_b) \times 100\% \quad (5)$$

where A_s represents the optical density value of the sample solution, and A_b represents the optical density value of the blank medium DMEM treatment.

Cellular model antioxidant activity of hydrogels

Mouse fibroblast NIH-3T3 was planted in a 96-cell well plate at a density of ~1×10^5 cells/mL and cultured in an incubator of 5% carbon dioxide and 37 °C for 24 h. Subsequently, remove the old medium and carefully wash twice with PBS. The cells were then stimulated with different concentrations of H_2O_2 for 24 h, and then cell activity was determined by MTT method. The concentration of H_2O_2 with a cell survival at about 50% was selected for hydrogel-to-cell oxidative stress defense test. Following the above method, H_2O_2 at the above assay concentration and sample solution with different concentrations were added into the well for co-incubation with cell for 24 h. The cells were then stained with DCFH-DA in the dark at 37 °C. Fluorescence intensity was determined by flow cytometry (BD FACSArica III. Flow Cytometer, BD biosciences). Intracellular fluorescence signals were observed by laser confocal microscopy (TCS SP8, Leica, Germany).

Cell migration experiment

Mouse fibroblast NIH-3T3 cells were cultured at a density of ~1.5×10^5 cells/mL in the culture chamber of the wound healing insert (ibidi, 81176) in an incubator at 5% carbon dioxide at 37 °C. The cells were cultured for 24 h to form a confluent monolayer. The wound healing insert was carefully removed with a sterile sedum and washed two times with sterile PBS to create a gap of 500 μm. The samples were dissolved in DMEM, filtered through a sterile filter, and then serially diluted into sample solutions with different concentrations. The DMEM medium without a sample was used as a control group, and FBS was added to all groups to make the final concentration reach 0.5%. 1 mL of sample solution was added to each cell culture dish. The cell migration status at 12 h, 24 h, and 48 h was observed and photographed by an inverted biomicroscope (MF52-M, Mshot), and the scratched area was analyzed using ImageJ software. The wound healing rate was calculated as follows:

$$\text{Wound healing rate (\%)} = [(S_0\text{-}S_t)/S_0] \times 100\% \quad (6)$$

where S_0 represents the initial scratch area, and S_t represents the scratch area at t hours.

In vitro hemostatic test

The hemostatic properties of hydrogels were evaluated using a mouse liver hemostatic model. Specifically, healthy male mice with uniform body weight (17-20 g) were taken and anesthetized by intraperitoneal injection of chloral hydrate. The mouse was fixed on a sterile operating table, the mouse liver was exposed through an abdominal incision, and the surrounding serum was carefully removed to prevent errors in calculating blood weights. An incision with a diameter of 5 mm was made on the left hepatic lobe of the mouse with a scalpel, the surgical plate was inclined at 30°, and a weighed filter paper with a diameter of 7 cm was placed under the liver. Different measures were taken after injury: the control group was not treated, and the sample treatment group was covered with commercial dressing and hydrogel. The amount of blood lost during the hemostasis process was recorded, and the time when blood loss stopped.

$$\text{Relative blood loss (\%)} = M_s/M_k \times 100\% \quad (7)$$

where M_s and M_k represent the blood loss weight of the sample and control groups, respectively. (n = 5).

Procoagulant test of hydrogels

The procoagulant properties of hydrogels were evaluated by the coagulation index (BCI) [1]. Specifically, fresh blood was drawn from mice and stored in a vacuum anticoagulant blood collection tube. 100 μL of the hydrogel precursor solution was added to a 10 mL sterile centrifuge tube, and then 45 μL of whole blood and 5 μL of calcium chloride solution (0.1 M) were added to activate the blood. Sterile deionized water was used as the reference for the hydrogel precursor. At different time points, sterile deionized water was added to dissolve the uncoagulated blood, and the OD value of the supernatant was measured at 540 nm. (n = 3). The coagulation index formula is as follows:

$$\text{BCI (\%)} = (OD_{sample}/OD_{water}) \times 100\% \quad (8)$$

Hemocompatibility assay of hydrogels

The hemocompatibility of the hydrogel was assessed by mouse red blood cell (RBC) suspension. The specific operation steps were as follows: fresh mouse whole blood and mouse erythrocytes were collected by centrifugation (2000 rpm, 10 minutes) with three times of PBS washing. The RBC suspension was subsequently diluted with PBS to a concentration of 5% (v/v) as a pre-made solution. 400 μL of RBCs precast solution and 50 μL of hydrogel were added to a 500 μL PCR tube and incubated at 37 °C for 3 h. PBS was used as a negative control, and water was used as a positive control. After that, the mixture was centrifuged (2000 rpm, 10 min), and the absorbance of the supernatant at 540 nm was tested with a microplate reader. The hemolysis rate was calculated according to the following formula:

$$\text{Hemolysis (\%)} = [(A_s - A_n)/(A_p - A_n)] \times 100\% \quad (9)$$

where A_s, A_n, and A_p represent the absorbance at 540 nm of the samples treated with the sample, negative control, and positive control groups, respectively. (n = 3)

Healing of hydrogels on full-layer skin defect models

The effect of hydrogels on wound healing was evaluated using a full-layer mouse skin model. With the approval of the Committee of the Center for Laboratory Animals of Jiangnan University, 60 female Balb/c mice weighing 17 to 20 g were randomly divided into four groups, blank control group, commercial Hydrosorb group, HQCS-SP group, and HQCS group (n = 5). Mice are anesthetized with 3% chloral hydrate. We then inflict a 0.7 cm circular full-layer skin defect on the back of the mouse with a 7 mm skin sampler. The hydrogels were covered in defect areas according to different groups, and the control group was not treated. Wound photographs were taken regularly, and during the measurement and analysis, a fine ruler was placed to calibrate the magnification of the wound area photographs. The defect area was calculated by measuring the wound area by Image J (National Institutes of Health).

$$\text{Wound closure rate (\%)} = [(S_0 - S_t)/S_0] \times 100\% \quad (10)$$

where S_0 and S_t represent the initial wound area and t-day wound area ($n = 5$), respectively. Mice were sacrificed on days 3, 7, and 14 after injury. Wound tissue was collected and fixed with 4% paraformaldehyde, paraffin-embedded. Tissue sections were subjected to hematoxylin-ebony (H&E) and Masson trichromatic staining, and the histopathological changes of the wound surface were observed under the panoramic slide scanner (Pannoramic DESK/MIDI/250/1000, 3DHISTECH). The collagen fiber content at each time point of each set of wound surfaces was calculated as follows:

$$\text{collagen fiber (\%)} = A_0 \text{ (type I collagen area)}/A_1 \text{ (total tissue area)} \times 100\% \quad (11)$$

5 zones were randomly selected for each set of specimens for analysis. The same method was used to determine the granulation and dermal tissue thickness. Histology and immunohistochemistry: regenerated skin specimens were excised on days 3 and 7, and tissue homogenizations were analyzed for levels of inflammatory factors IL-6 and IL-10 by Mouse IL-6 ELISA Kit, Mouse IL-10 ELISA Kit (Multisciences Biotech Co., Ltd).

Statistical analyses

All the experimental data were statistically analyzed as a mean ± standard deviation (SD). The data were analyzed by one-way ANOVA followed by Tukey's test (GraphPad Prism 8).

Results and Discussions

As shown in Figure S1a, in the FTIR of QCS, the presence of a secondary amide was demonstrated by the observation of the C=O telescopic vibration peak near 1631 cm^{-1} and the NH curved vibration peak and the C-N telescopic vibration peak (two peaks coincident) near 1529 cm^{-1}. Characteristic absorption peak at 1470 cm^{-1} methyl bond designated as GTMAC in QCS. UV absorption indicates that a significant catechol absorption peak appears at 280 nm, indicating that dihydrocaffic acid has successfully grafted onto quaternary ammonium hydrocarbon glycan (Figure S1b). According to the test concentration combined with HCA indexing analysis, the grafting rate of catechol was determined. At the same time, according to the ^1H NMR hydrogen spectroscopy test, the peak at 6.6-6.9 ppm belongs to the catechol group (Figure S1c). The characteristic peaks at 3.2 ppm and 3.4 ppm belong to the trimethylammonium group and the –NH-CH_2- group, respectively. This result confirmed the successful synthesis of catechol modified quaternary ammonium chitosan (QCS-C). The degree of grafting of quaternary ammonium on chitosan is 67.8%, and the degree of conjugation of catechol is 16.7%, which is calculated by the integral value in NMR spectrum. UV-Vis spectra confirmed that catechol was successfully grafted onto QCS through the absorption peak at 280 nm and the degree of conjugation of catechol is 22.3%.

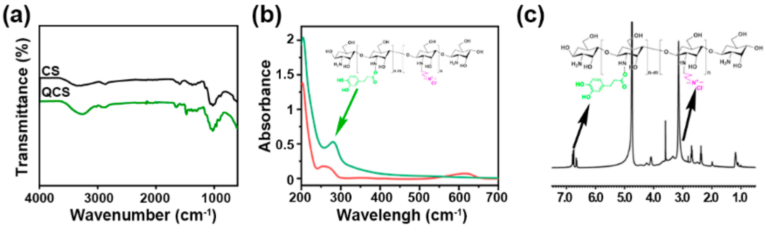

Figure S1. Characterization of HQCS-SP hydrogel. (**a**) FTIR of CS and QCS. (**b**) UV-vis spectrum: absorbance of 0.1% HQCS and 0.1% SP in the range of 200-700 nm. (**c**) Nuclear magnetic hydrogen spectroscopy of HQCS.

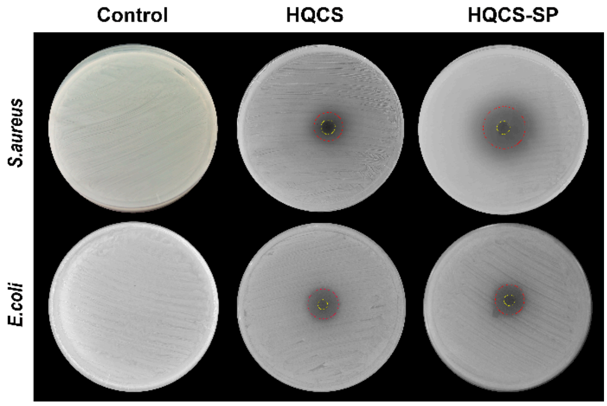

Figure S2. Effect map of the bacteriostatic circle of the hydrogel on the target bacteria.

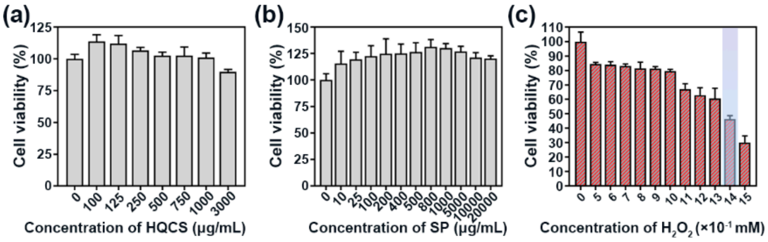

Figure S3. NIH-3T3 cell viability. (**a**) and (**b**) are NIH-3T3 cell activities at different concentrations of HQCS and SP. (**c**) Cellular oxidative stress model: survival rate of cells under H_2O_2 at different concentrations.

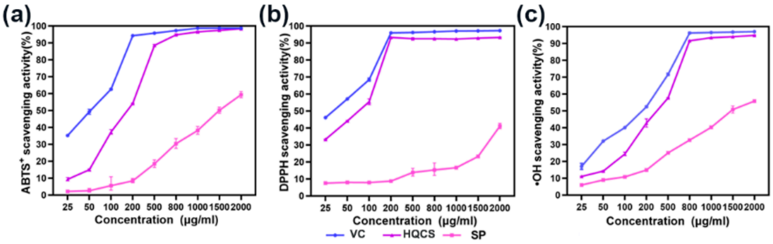

Figure S4. The ABTS, DPPH and OH radical scavenging activities of HQCS, SP, and positive control VC at different concentrations.

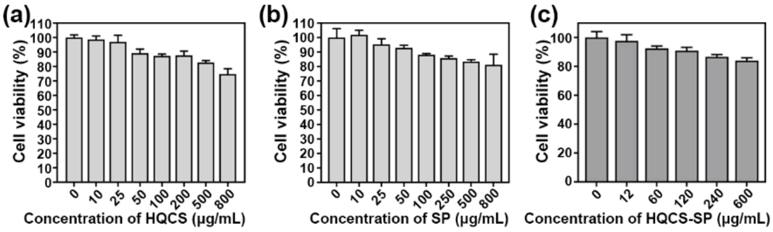

Figure S5. RAW 264.7 cell activities at different concentrations of HQCS, SP and HQCS-SP.

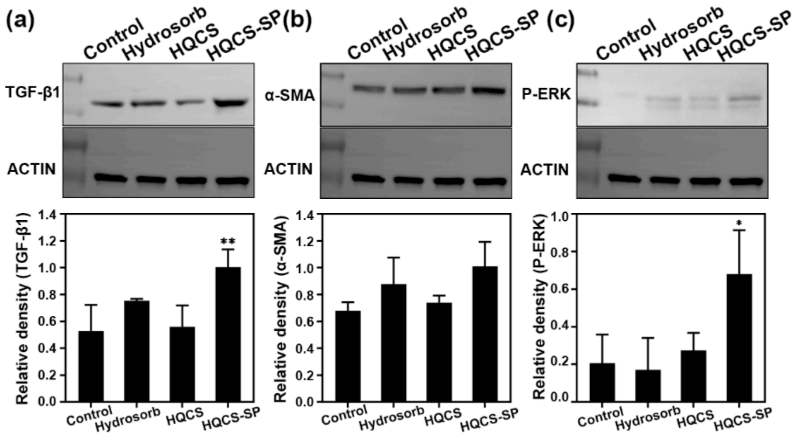

Figure S6. Western blot results. (**a**) The protein expression level of TGF-β1 was measured by western blot analysis following different treatment for 7 days. (**b**) The expression level of α-SMA was measured by western blot analysis following different treatment for 7 days. (**c**) The phosphorylation level of ERK was measured by western blot analysis following different treatment for 7 days. Each value represents the mean ± standard deviation of three independent experiments. *$p < 0.05$, **$p < 0.001$ compared to the control group.

HQCS-SP through TGF-β1 signal pathway enhances wound repair. TGF-β1 signal transduction pathway plays an important role in tissue repair. TGF-β1 involve in the whole process of inflammation, proliferation and plasticization during wound repair. Therefore, TGF-β1 was determined by western blot analysis signal path. The results showed that TGF-β1 in skin granulation tissue of mice treated with HQCS-SP (Figure S6a). These results indicated that HQCS-SP passes TGF-β1 signal pathway promotes skin wound healing in mice.

Myofibroblast plays an important role in skin wound repair. One of the specific markers of myofibroblast is α-SMA. Therefore, in this study, western blot analysis was used to determine the expression level of α-SMA. The expression level of α-SMA of HQCS-SP was higher than that of control group (Figure S6b). These results suggested that HQCS-SP enhances wound healing by increasing the level of myofibroblasts.

HQCS-SP enhances wound repair in mice through ERK signaling pathway. According to previous research results on ccd-986sk cells [2], it is known that EGFR/ERK signaling pathway is involved in the induced proliferation and migration of ccd-988sk cells [3]. Therefore, the effect of HQCS-SP on ERK phosphorylation was determined by Western blot analysis. The results showed that the phosphorylation level of ERK in skin granulation tissue of mice treated with HQCS-SP increased (Figure S6c). This suggests that HQCS-SP promotes skin wound healing in mice through ERK signaling pathway.

References

1. Teng, L.; Shao, Z.; Bai, Q.; Zhang, X.; He, Y.S.; Lu, J.; Dong, C.M. Biomimetic glycopolypeptide hydrogels with tunable adhesion and microporous structure for fast hemostasis and highly efficient wound healing. *Adv. Funct. Mater.* **2021**, 31, 2105628.
2. Liu, P.; Lee, M.K.; Choi, J.W.; Choi, Y.H.; Nam, T.J. Crude protein from spirulina increases the viability of CCD-986sk cells via the EGFR/MAPK signaling pathway. *Int. J. Mol. Med.* **2019**, 43, 771-778.
3. Liu, P.; Choi, J.W.; Lee, M.K.; Choi, Y.H.; Nam, T.J. Wound healing potential of spirulina protein on CCD-986sk cells. *Mar. Drugs* **2019**, 17, 130.
